# A Peak of H3T3 Phosphorylation Occurs in Synchrony with Mitosis in Sea Urchin Early Embryos

**DOI:** 10.3390/cells9040898

**Published:** 2020-04-07

**Authors:** Omid Feizbakhsh, Florian Pontheaux, Virginie Glippa, Julia Morales, Sandrine Ruchaud, Patrick Cormier, Fernando Roch

**Affiliations:** Centre National de la Recherche Scientifique (CNRS), Sorbonne Université, Integrative Biology of Marine Models (LBI2M), Station Biologique de Roscoff, CS 90074, 29680 Roscoff CEDEX, France

**Keywords:** histone phosphorylation, Haspin, mitosis, cell division, CDK1, CHR-6494, Roscovitine, fertilisation, sea urchin embryo, *Sphaerechinus granularis*

## Abstract

The sea urchin embryo provides a valuable system to analyse the molecular mechanisms orchestrating cell cycle progression and mitosis in a developmental context. However, although it is known that the regulation of histone activity by post-translational modification plays an important role during cell division, the dynamics and the impact of these modifications have not been characterised in detail in a developing embryo. Using different immuno-detection techniques, we show that the levels of Histone 3 phosphorylation at Threonine 3 oscillate in synchrony with mitosis in *Sphaerechinus granularis* early embryos. We present, in addition, the results of a pharmacological study aimed at analysing the role of this key histone post-translational modification during sea urchin early development.

## 1. Introduction

Fertilisation is not just the encounter of two gametes; it is also a decisive event triggering the transformation of a quiescent egg into an actively developing embryo. Sea urchins constitute a powerful model system to study the molecular mechanisms orchestrating this radical conversion, which is a key step in the early life of all metazoan organisms [1]. In sea urchins, mature eggs are haploid cells arrested in the G1-phase of the cell cycle. As meiosis has been completed before fertilisation, sperm entry can be thus regarded as a proliferative signal promoting cell division. In addition, mRNA transcription and ribosome biogenesis seem largely dispensable for the three first mitotic divisions [2,3]. From a mechanistic perspective, this implies that cell cycle progression relies on the temporal and spatial control of maternal mRNA translation and a complex cascade of post-translational modifications. Consistently, the dramatic rise in mRNA translation observed in fertilised eggs has been shown to be essential for M-phase completion [4,5], and the contribution of protein phosphorylation to the first mitotic cell cycles has been recognised as crucial [6].

Cell division is the result of a complex series of coordinated events, including chromatin condensation, formation of a bipolar spindle, chromosome alignment and separation of sister chromatids [7]. The Cyclin B/CDK1 (Cyclin Dependent Kinase 1) complex is the key factor orchestrating these different processes, acting upstream of a cascade of post-translational regulatory events [8]. For instance, the contribution of this complex is essential for the activation of the multiple kinases and phosphatases participating during kinetochore assembly and chromosome segregation [9,10]. However, despite the fact that Cyclins were first described in sea urchin embryos [11], little is known in these animals about the identity of the Cyclin B/CDK1 targets, especially at the chromatin level. In fact, although the regulation of histone activity via post-translational modifications is likely to play a central role during chromatin condensation and chromatid separation [12,13], the dynamics of these modifications and their functional significance have not been studied in depth during sea urchin development [14].

The presence of high levels of phosphorylated histone H3 is one of the unequivocal signatures of cell division, and it is well established that the H3 Ser10 and Ser28 residues are phosphorylated during chromosome condensation in multiple species [15,16,17]. However, the H3 also becomes phosphorylated at Thr3 during mitosis in many different taxa, including mammals [18,19], insects [20], plants [21] and fungi [22]. In mammalian cells, the levels of the T3H3 phosphorylated form (T3H3ph) oscillate following a strict temporal and spatial pattern. T3H3ph appears during early prophase in areas of condensed chromatin, occupies the pericentromeric chromatin and the chromosome arms during prometaphase and metaphase, and disappears by late anaphase [18,19].

The Haspin protein (Haploid Germ Cell-specific Nuclear Protein Kinase) was originally characterised in mammalian cells as the main kinase responsible for T3H3 phosphorylation during mitosis [18,23]. In addition, different Haspin homologs have been described in animals, plants and fungi, indicating that this kinase appeared early during eukaryotic evolution [24,25]. Interestingly, H3T3 phosphorylation also depends on the activity of a Haspin kinase in the fission yeast *S. pombe* [22] and the plant *A. thaliana* [26,27], suggesting that this protein could participate in a highly conserved regulatory circuit.

The use of genetic tools and the discovery of specific inhibitors targeting Haspin have contributed decisively to the functional characterisation of this kinase, offering in parallel an opportunity to study the biological significance of H3T3 phosphorylation [28]. Haspin depletion by RNA interference in mitotically active cells results in low levels of T3H3ph and promotes the accumulation of cells assuming a late prometaphase aspect [18]. Another study has shown that Haspin depletion prevents normal chromosome alignment and thus activates the spindle assembly checkpoint [29]. In addition, Haspin-depleted mitotic cells show extra centrosome-like foci that nucleate microtubules [30]. Therefore, Haspin is required for the normal alignment of chromosomes, participates in the regulation of kinetochore assembly and contributes to the formation of bipolar spindles. Moreover, the use of Haspin inhibitors has shown that this kinase is also required during meiosis [31,32]. However, much remains to be done, as Haspin is likely to play a biological role also during interphase [33] and H3 might not be its sole relevant substrate [34,35].

In this study, we examined the dynamics and the localisation of T3H3ph during the first cleavage of the *Sphaerechinus granularis* embryo. We showed that sea urchins have a single Haspin homolog and we found that their first mitotic division is strongly delayed in the presence of the Haspin CHR-6494 inhibitor (Huertas et al., 2012). However, our results indicate that this drug could be targeting the activity of the Cyclin B/CDK1 complex. Our observations are nevertheless consistent with the idea that T3H3 phosphorylation is tightly coupled to cell cycle progression and Cyclin B/CDK1 activity during the early embryogenesis of sea urchins.

## 2. Materials and Methods

### 2.1. Antibodies and Reagents

To detect the T3H3ph form, we used the monoclonal antibody JY325 (Merck) directed against the human phosphorylated Histone 3.1 variant. The human and sea urchin Histone 3 homologs present virtually identical sequences (Figure S1a), and this antibody detects a ~17 kDa single band in *S. granularis* embryonic protein extracts (Figure S1b). Other antibodies used include anti-PP1Cα (ab66955; Abcam), anti-Actin (20-30, Sigma-Aldrich France), anti-H3 (865R2, Invitrogen), anti-CDK1 PSTAIR (P7962, Sigma-Aldrich France) and anti-Tubulin ascites 3F3 (a kind gift of Chloë Bulinsky, Columbia University, New York). We also used the anti-PP1CαT320ph (ab62334, Abcam) to detect the corresponding T318PP1Cph form in *S. granularis* [36]. CHR-6494, Roscovitine and other chemicals used were purchased from Sigma-Aldrich France, unless otherwise stated.

### 2.2. Handling of Gametes and Embryos

*S. granularis* adult specimens collected in the Brest area (France) were supplied by the CRBM (Centre de Ressources Biologiques Marines) of the Roscoff Biological Station. Gamete spawning was induced by intracoelomic injection of 0.1 M acetylcholine and eggs were raised in 0.22 µm Millipore-filtered seawater (FSW). Eggs were de-jellied for one minute in 3.5 mM citric acid pH 5 and rinsed subsequently three times with fresh FSW. For fertilisation, a 2.5% *v*/*v* egg suspension was mixed with freshly diluted dry sperm. Fertilisation rates observed were always higher than 90% and experiments run in parallel were performed using the gametes of a single female. Embryo cultures were maintained under constant agitation in FSW at 16 °C. When required, CHR-6494, Roscovitine or DMSO were added at different concentrations and time points, as indicated.

### 2.3. Quantification of Cleavage Rates and Phenotypic Analysis

Live embryos were inspected with a phase-contrast microscope at regular intervals during the 5 h following fertilisation. For each time point, the proportion of cleaved eggs was determined in a random sample containing roughly a hundred embryos. For analysis of immunostained samples, we defined five phenotypic categories corresponding to the different stages of mitosis: Prophase; Prometaphase; Metaphase; Anaphase and Telophase. Their relative proportions were estimated in each sample by visually inspecting *n* ≥ 233 embryos in a series of randomly chosen optical fields, using a Leica fluorescence microscope.

### 2.4. Embryo Protein Extracts and Western Blot Analyses

We collected 500 μL samples from each embryo culture at different time points. Embryos were subsequently pelleted by quick centrifugation, immediately frozen in liquid nitrogen and kept at −80 °C. Cell lysates were obtained by incubating pellets for 10 min at 4 °C in 100 µL of a buffer containing 50 mM Tris (pH 8), 250 mM NaCl, 1 mM EDTA (pH 8), 1% Triton X-100, 1% Sodium-Deoxycholate, 1% Sodium Dodecyl-sulfate (SDS), 1/100 phenylmethylsulfonyl fluoride (PMSF) and a 1/1000 dilution of a protease inhibitor cocktail containing 100 mM 4-[2-aminoethyl] benzenesulfonyl fluoride hydrochloride (AEBSF), 800 mM aprotinin, 50 mM bestatin, 15 mM E-64, 20 mM leupeptin and 10 mM pepstatin. After HCl addition to a final concentration of 0.2 N, cell lysates were incubated for 30 min at 4 °C and then centrifuged at 12,000× *g* for 10 min at 4 °C. Supernatants were transferred into new tubes and acid neutralisation was performed adding NaOH to a final concentration of 0.2 N. Then, 125 µL of 2× loading buffer were added (NuPAGE^TM^ LDS Sample Buffer 4× and 200 mM DTT). Proteins were resolved by SDS-PAGE on 12% acrylamide gels and their levels analysed by Western blot, using the T3H3ph (1/8000), H3 (1/1000), Actin (1/1000), T318PP1Cph (1/1000) and PP1C (1/10,000) antibodies. We used horseradish peroxidase-coupled secondary antibodies (Dako SA) and the ECL kit (Thermo-Fisher) to produce a signal that was detected with a Fusion FX camera (Vilber-Lourmat). Results were quantified using the ImageJ software (written by Wayne Rasband at the US National Institute of Health, Bethesda, Maryland, USA, http://imagej.nih.gov/ij/, 1997–2015.).

### 2.5. Immunostaining and Confocal Microscopy

We followed a protocol specifically developed to image the cytoskeleton in echinoderm embryos [37]. Briefly, we collected 1.6 mL samples from each embryo culture at the indicated times. Embryos were rinsed with 1.8 mL of a solution containing 80 mM PIPES (pH 7.2), 5 mM EGTA, 5 mM MgCl_2_ and 1 M glycerol, and immediately fixed for 1 h at room temperature in 2 mL of the same solution supplemented with 3.7% formaldehyde and 0.1% Igepal CA630. Subsequently, embryos were rinsed three times with 1× PBS, 0.1% Triton X-100 (PBT). Blocking for 1 h, overnight primary antibody incubation at 4 °C and subsequent washings were carried out in PBT supplemented with 1% BSA. We used as primary antibodies T3H3ph and anti-Tubulin 3F3, both at a 1/600 dilution. Goat anti-rabbit AlexaFluor 555 and donkey anti-mouse AlexaFluor 488 (Invitrogen) were used as secondary antibodies, diluted 1/1000 in PBT 1% BSA. After incubation for 2 h in the dark, embryos were washed three times for 45 min in PBS and mounted in a drop of Vectashield^®^ medium (Vector) containing DAPI. Images were acquired with a Leica SP5 confocal microscope and processed using ImageJ and Photoshop.

## 3. Results

### 3.1. The Levels of T3H3ph Cycle in Synchrony with Cell Division in Sea Urchin Early Embryos

**Figure 1:**
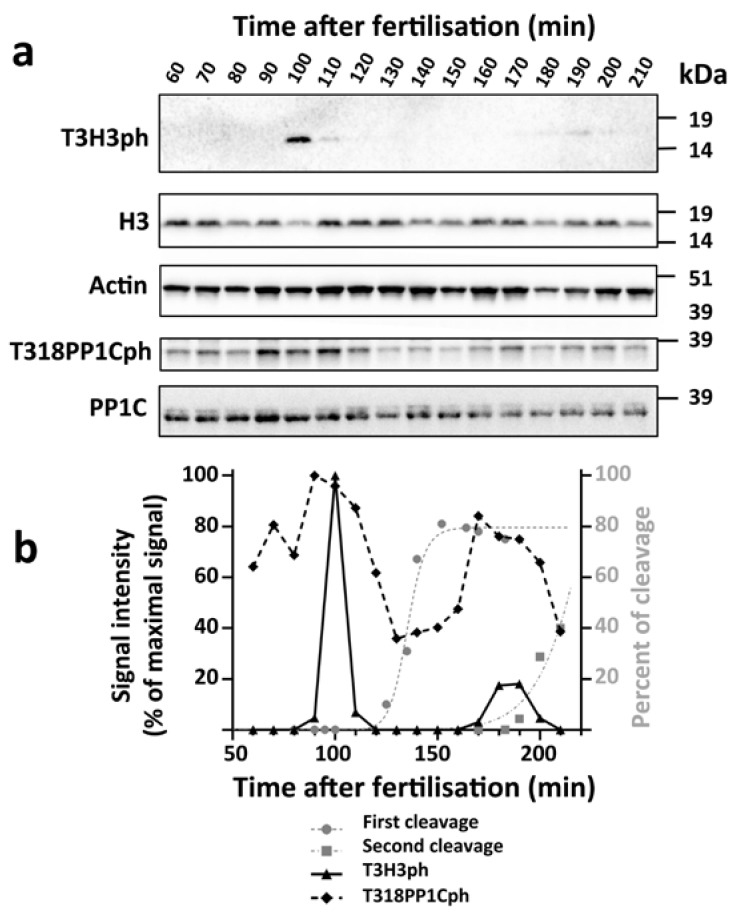
A peak of T3H3 phosphorylation precedes cell cleavage in *S. granularis* early embryos. (**a**) Western blots showing the relative levels of T3H3ph, H3, Actin, T318PP1Cph and PP1C at different times during the first and second embryonic divisions. (**b**) Quantitative analysis of the above results. For each lane, the T3H3ph (black triangles) and T318PP1Cph (black diamonds) intensity scores were normalised against the amount of Actin signal detected. The values obtained were expressed as a percentage of the maximal signal observed. The graph includes as a visual reference the proportion of embryos that have completed either the first cleavage (grey dots) or the second cleavage (grey squares) at different time points.

First, we sought to establish if the levels of T3H3ph oscillate during the early embryogenesis of *S. granularis*. For this, embryo protein extracts were prepared at different times post-fertilisation (PF) and analysed by Western blot, taking advantage of an antibody recognising specifically the T3H3 phosphorylated form (see Materials and Methods and Figure S1). The T3H3ph signal was first detected at 90 min PF, reached its maximal levels at 100 min PF and decayed abruptly by 110 min PF (Figure 1). This peak of T3H3 phosphorylation was followed by a second one taking place at 180–190 min PF (Figure 1).

We observed that both T3H3ph peaks occurred roughly 30 min before half of the embryos completed cell cleavage (Figure 1b). Accordingly, and despite the fact that the precise timing of cell division can vary in the progeny of different individuals, we found similar dynamics in the offspring of other *S. granularis* couples (Figure S2). The observed fluctuations in the T3H3ph signal correspond to a post-translational modification of H3, as we observed that the levels of this protein remained stable during early embryonic development (Figure 1a and Figure S2). We also examined in the same embryo cultures the temporal activation profile of the Cyclin-dependent kinase CDK1 (Figure 1 and Figure S2). One of the substrates of this key cell cycle regulator is the PP1C phosphatase [38]. In sea urchin embryos, this protein becomes phosphorylated at T318 and its levels can thus provide a read-out of the CDK1 global activity [36]. We observed a peak of T318PP1Cph at 90 min PF, prior to the first embryonic division, and another one at 170 min PF, before the second division (Figure 1). In contrast, the levels of PP1C displayed little variation during the examined period (Figure 1a). Altogether, our observations indicate that cell cleavage is preceded in *S. granularis* embryos by a wave of CDK1 activity and a sharp peak of T3H3 phosphorylation.

**Figure 2:**
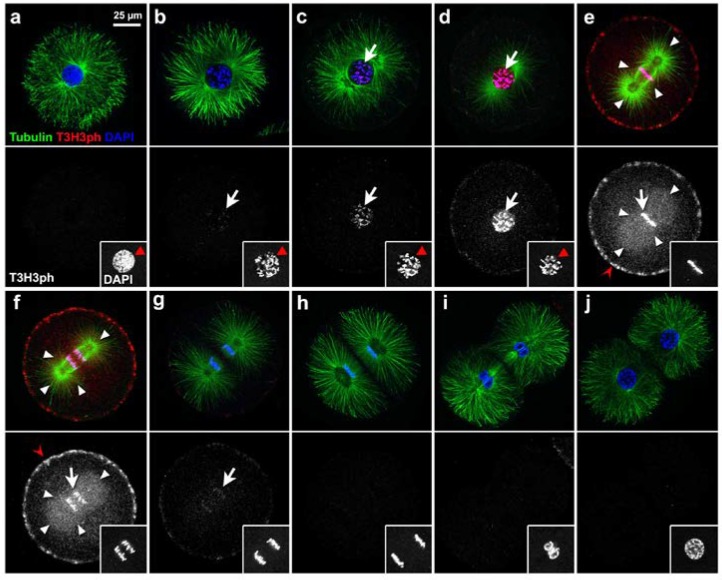
A wave of T3H3 phosphorylation/de-phosphorylation occurs in synchrony with mitotic division in *S. granularis* early embryos. (**a**–**j**) Pictures correspond to confocal equatorial optical sections obtained by imaging embryos immunostained for Tubulin (shown in green, top panels) and T3H3ph (red in the top panels, greyscale in bottom panels). Samples were also labelled with DAPI (blue in top panels, grey-scale in small insets). Images present consecutive stages illustrating progression through the first embryonic division. Scale bar, 25 µm. (**a**–**d**) The T3H3ph signal (white arrows) appeared when chromatin condensation was already apparent in the cell nucleus (red arrowheads, insets). (**e**,**f**) A strong T3H3ph signal (white arrows) was observed in association with chromatin, both during the metaphase (**e**) and early anaphase (**f**). In these stages, a fluorescent signal associated with the cell cortex (red open arrowheads) and the mitotic spindle (white arrowheads) was also visible. (**g**–**h**) During the anaphase, the T3H3ph signal (white arrows) rapidly faded away. (**i**,**j**) No T3H3ph signal was detected during the telophase.

To further confirm that T3H3 phosphorylation oscillates in synchrony with cell division, we also studied the spatio-temporal distribution of T3H3ph in immunostained embryos. To analyse the first embryonic division, fertilised eggs were recovered at 70, 110 and 150 min PF and co-labelled with Tubulin antibodies and the nuclear marker DAPI, to allow simultaneous visualisation of their cytoskeletal architecture and their chromatin organisation (Figure 2).

**Figure 3:**
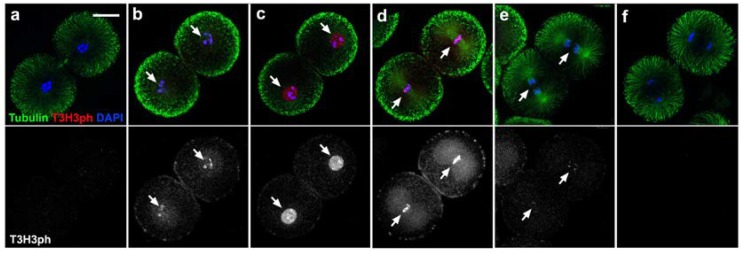
A peak of T3H3 phosphorylation accompanies the second mitotic division in *S. granularis* embryos. (**a**–**f**) Pictures correspond to confocal equatorial optical sections obtained by imaging embryos immunostained for Tubulin (shown in green, top panels), T3H3ph (red in the top panels, greyscale in bottom panels) and DAPI (blue in top panels). Images present consecutive stages illustrating progression through the second embryonic division. Scale bar, 30 µm. (**a**,**b**) The T3H3ph signal (white arrows) appeared during chromatin condensation. (**c**,**d**) A strong T3H3ph signal (white arrows) was observed during the prometaphase (**c**) and metaphase (**d**). (**e**,**f**) The T3H3ph signal (white arrows) disappears during the anaphase.

During mitosis, chromatin condensation was accompanied by a thorough reorganisation of the microtubule network, which culminated by prometaphase with the formation of the spindle apparatus (Figure 2a–d). The T3H3ph signal was first detected during this condensation phase, in small foci associated with the forming chromosomes (Figure 2b–d). Later on, chromatin acquired a uniform and bright T3H3ph staining characteristic of cells in prometaphase (Figure 2d and Figure S3). We also detected high levels of T3H3ph throughout the metaphase (Figure 2e and Figure S3), but by early anaphase, signal intensity dropped abruptly and became undetectable (Figure 2f–j). Besides the T3H3ph signal detected at the chromatin level, we also observed during the metaphase and anaphase a conspicuous staining associated with the egg’s cortex and a central region occupied by the mitotic spindle (Figure 2e,f). Whether this signal reflected the presence of H3 in these subcellular compartments or was the result of an unspecific binding of our antibody could though not be determined. Finally, we analysed embryos harvested at 160, 180 and 200 min PF and observed that the levels of T3H3ph also peak during the second mitotic division (Figure 3). Altogether, our results confirm that a wave of T3H3 phosphorylation/dephosphorylation accompanies cell division in *S. granularis* early embryos.

### 3.2. The Haspin Inhibitor CHR-6494 Delays Entry into Mitosis

**Figure 4:**
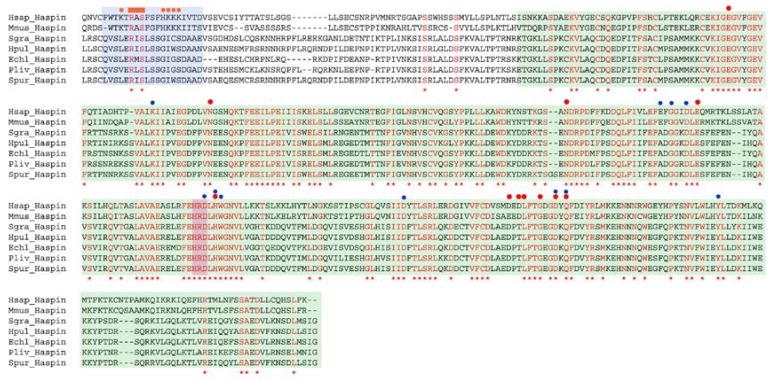
The echinoidea Haspin homologs display a conserved Ser/Thr kinase domain. Clustal-W protein alignment showing the C-terminal portion of Haspin in different mammalia and echinoidea species. Invariant amino acid positions are shown in red and labelled with asterisks. The Haspin Ser/Thr kinase domain is shaded in green and its HRD catalytic loop is shown in red. Residues implicated in H3 recognition and/or interacting with ATP/Mg^2+^ in the human Haspin are respectively marked with red and blue dots [34,39,40]. The vertebrate HBIS motif and its homolog region in sea urchins are shaded in blue. The positively charged residues of the vertebrate motif are marked with orange dots [42], but do not appear to be conserved. The RAS motif, where the Ser is one of the residues phosphorylated by the Polo-kinase during Haspin activation is indicated by an orange rectangle [42]. We aligned the following sequences: Hsap, *Homo sapiens* (NP_114171.2); Mmus, *Mus musculus* (NP_038578.2); Spur, *Strongylocentrotus purpuratus* (JT120475.1); Sgra, *Sphaerechinus granularis* (GAVR01006044.1) and (GAVR01015122.1); Echl, *Evechinus chloroticus* (GAPB01047772.1); Pliv, *Paracentrotus lividus* (GEDS01040703.1) and Hpul, *Hemicentrotus pulcherrimus* (IACU01096736.1).

It has been shown that the activity of the Haspin kinase is essential for T3H3 phosphorylation in different animal, yeast and plant species [18,22,26,27]. Taking advantage of available transcriptome databases, we conducted a search for Haspin representatives among the echinoidea and identified a single homolog of this gene in several sea urchin species (Figure 4). The echinoidea Haspin proteins harbour in their C-terminus a Ser/Thr kinase domain displaying the typical features of Haspin kinases. Indeed, most of the residues known to be involved in substrate recognition in the human protein [34,39,40] are conserved in the sea urchin Haspin homologs (Figure 4). These structural similarities prompted us to examine whether Haspin is implicated in T3H3 phosphorylation during the *S. granularis* first cell division. For this, we used the Haspin inhibitor CHR-6494 [41]. This small molecule behaves as an ATP competitor and is thought to act at the level of the Haspin catalytic cleft [41]. Moreover, CHR-6494 administration is known to prevent in vivo T3H3 phosphorylation in different species [31,32,41].

**Figure 5:**
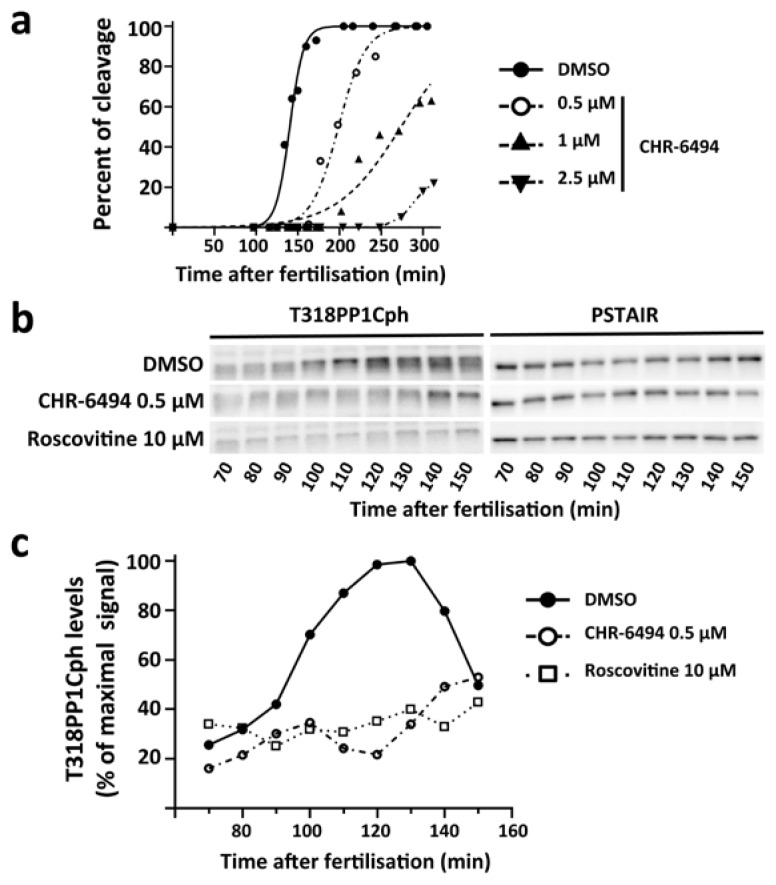
Exposure to the CHR-6494 inhibitor delays cell division in *S. granularis* early embryos. (**a**) Graph representing the proportion of embryos that have completed the first cleavage at different time points (min after fertilisation). The different curves describe culture behaviour in the presence of increasing concentrations of CHR-6494 or its vehicle DMSO, all added at 15 min after fertilisation. (**b**) Relative levels of T318PP1Cph observed at different time points in embryos treated with DMSO, 0.5 µM CHR-6494 or 10 µM Roscovitine. The T318PP1Cph form was detected by Western blot using a specific antibody. The levels of CDK1 were visualised in the same membrane with the PSTAIR antibody and used as a loading control. (**c**) Quantitative analysis of the above results. Plotted values were normalised against the CDK1 levels and expressed as a percentage of the maximal signal observed.

Fifteen minutes after fertilisation, we added increasing concentrations of CHR-6494 to different batches of the same embryo culture. We observed that drug addition delayed cell cleavage in a dose-dependent manner (Figure 5a). Moreover, the levels of the T318PP1Cph marker increased significantly during mitosis in control batches (Figure 5b,c), but failed to do so in the presence of 0.5 µM CHR-6494 (Figure 5b,c). Therefore, CHR-6494 addition hinders cell cycle progression in *S. granularis* embryos.

Our observations indicate that Haspin could play a major role during the *S. granularis* first cleavage, but we cannot rule out the possibility that CHR-6494 may inhibit in vivo the activity of other kinases involved in cell cycle progression, like CDK1 [41]. In fact, the CDK1 inhibitor Roscovitine [43,44] elicited a reduction in the T318PP1Cph levels comparable to that observed upon CHR-6494 addition (Figure 5b,c and [36]). We should nonetheless stress that in these experiments both CHR-6494 and Roscovitine were administered way before the Cyclin B/CDK1 activity could attain its maximal levels (Figure 1 and Figure 5). Therefore, we wondered if the same inhibitors could elicit different effects if added after the initial Cyclin B/CDK1 activation phase, in particular immediately before T3H3 becomes phosphorylated. Such an assay could provide an opportunity to compare the effects of CHR-6494 with those resulting from a genuine CDK1 inhibition, thus allowing a better assessment of CHR-6494 specificity.

**Figure 6:**
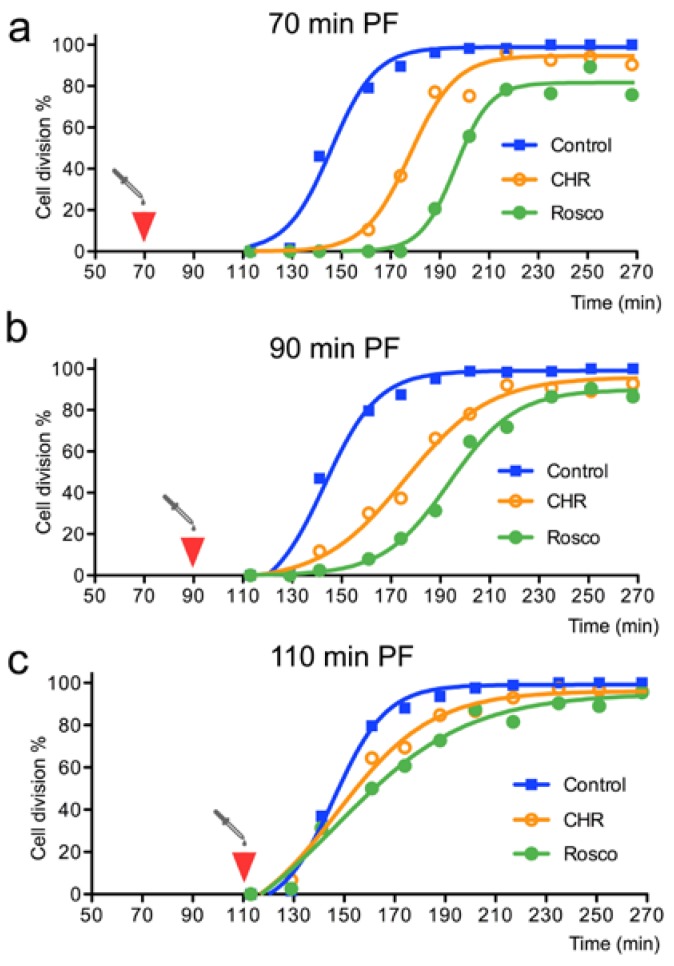
CHR-6494 and Roscovitine administration delays cell division and impacts cleavage rates in *S. granularis* embryos. (**a**–**c**) Plots represent the proportion of embryos that have completed their first cleavage at different time points (minutes PF). Final concentrations for CHR-6494 and Roscovitine were respectively 0.5 µM and 10 µM. For each experimental condition, a culture sample was collected 20 min after drug addition and processed for T3H3ph immunostaining (see Figure 7). (**a**) Drug addition at 70 min PF (red arrowhead) caused a general delay in the cleavage onset. (**b**) Drug addition at 90 min PF delayed cleavage onset and perturbed culture synchronicity. (**c**) No delay in cleavage onset was observed, but culture synchronicity was still perturbed in embryos treated at 110 min PF.

**Figure 7:**
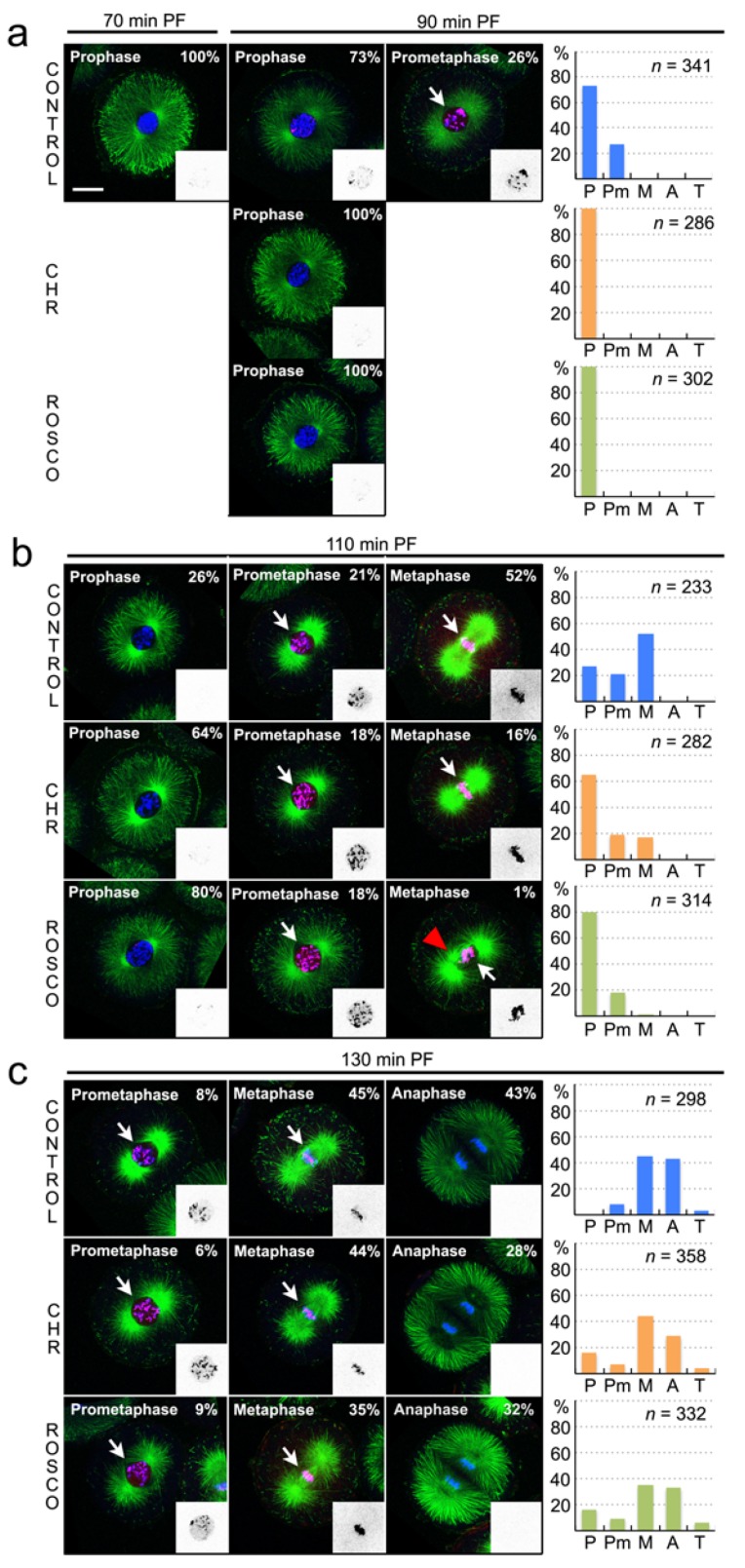
Neither CHR-6494 nor Roscovitine prevent T3H3 phosphorylation during the first embryonic division in *S. granularis*. (**a**–**c**) Embryos were assigned to five different phenotypic categories, according to their morphology. For each sample, representative images illustrating the different phenotypes found are shown in the same row. Pictures correspond to confocal equatorial optical sections obtained imaging embryos immunostained for Tubulin (shown in green), T3H3ph (red and inverted grey-scale in the small insets) and DAPI (blue). Scale bar, 25 µm. The relative proportions of the different categories are reported on each image and have been plotted as a series of histograms shown on the right panel of each row. The number of embryos considered for the quantitative analyses is also shown on the corresponding graphs. Abbreviations used: P, Prophase; Pm, Prometaphase; M, Metaphase; A, Anaphase; T, Telophase. (**a**) All embryos were in prophase in a sample collected before drug addition, at 70 min PF. In the control batch, samples collected at 90 min PF included 26% of embryos in prometaphase, all displaying high levels of T3H3ph (white arrow). This phenotypic category was absent in the drug-exposed batches. (**b**) Samples collected at 110 min PF contained embryos in prometaphase and metaphase, all showing a bright T3H3ph staining (white arrows). In the batch exposed to Roscovitine, all the embryos in metaphase presented ectopic spindle poles (red arrowhead). (**c**) In the 130 min PF samples, embryos in prometaphase and metaphase displayed normal levels of T3H3ph (white arrows) and the staining disappeared during the anaphase.

Embryos belonging to a synchronised culture were distributed in nine different batches and the two drugs were added separately at 70, 90 and 110 min PF. Then, the behaviour of each resulting cohort was analysed using two different methods. First, we compared the cleavage rates observed in each assay (Figure 6). Second, we monitored cell cycle progression by inspecting embryo morphology (Figure 7). For this, a sample was collected from each cohort 20 min after drug addition and immediately processed for immunostaining and confocal imaging, using Tubulin, T3H3ph and DAPI as cellular markers (Figure 7).

When drugs were added at 70 min PF, embryos exposed to either 0.5 µM CHR-6494 or 10 µM Roscovitine reached the cleavage stage with an obvious delay (Figure 6a). However, these batches exhibited the same degree of synchronicity observed in the corresponding control, and embryos proceeded eventually through cleavage at a similar pace (Figure 7a). A comparison of the corresponding immunostained samples revealed that 26% of the control embryos had reached the prometaphase stage by 90 min PF (Figure 7a). As expected, these cells displayed high levels of the T3H3ph marker (Figure 7a). In contrast, all the cells exposed to the inhibitors were in prophase and presented a similar morphology to that observed in embryos harvested right before drug addition, at 70 min PF (Figure 7a). Moreover, no cell presenting a robust T3H3ph signal could be observed in these samples (Figure 7a).

When drugs were added at 90 min PF, cleavage onset was also delayed. However, this treatment seemed to perturb culture synchronicity as well (Figure 6b). We could estimate this parameter measuring the time elapsed between cleavage onset and the moment in which 90% of the embryos completed cell division. This interval lasted for 59 min in a control population and 56 min when CHR-6494 was added at 70 min PF. However, in embryos treated with the same drug at 90 min PF, it lasted for 94 min (Figure 6a,b). Therefore, sample dispersion increased when drugs were added at 90 min PF. In these batches, immunostained samples were collected at 110 min PF. We observed again that the cells in prophase were overrepresented in the samples treated with CHR-6494 or Roscovitine (Figure 7b). Moreover, 52% of the embryos were in metaphase in the control population, whereas only 16% and 1% of the cells had respectively reached this stage in cultures exposed to CHR-6494 or Roscovitine (Figure 7b). Surprisingly, all cells exposed to the drugs presented normal T3H3ph levels during the prometaphase and metaphase (Figure 7b). However, we noticed that the mitotic apparatus assumed an aberrant morphology during the metaphase in all the cells treated with Roscovitine. Indeed, our images revealed in these embryos the presence of ectopic spindle poles (Figure 7b).

Finally, we analysed cell behaviour upon drug addition at 110 min PF (Figure 6c and Figure 7c). In these batches, drug exposure did not delay cleavage onset significantly, but culture synchronicity was again perturbed (Figure 6c). In fact, although virtually no prophase embryos could be found in the control group at 130 min PF, this category was well represented in cultures exposed to either CHR-6494 or Roscovitine (Figure 7c). Besides this, the embryos in prometaphase and metaphase displayed comparable levels of T3H3ph in all the conditions analysed. In addition, no obvious aberrant mitotic figures could be detected (Figure 7c).

In summary, our data show that CHR-6494 and Roscovitine affect cell cycle progression in a similar way. Both inhibitors delay cleavage onset and seem to act primarily by retarding entry into prometaphase. However, drug presence does not directly impact the levels of T3H3ph when cells reach the prometaphase or posterior stages.

## 4. Discussion

Although it has been shown that the levels of T3H3ph cycle during mitosis in different somatic and germ line cells, the phosphorylation state of T3H3 has not been examined during the early embryogenesis of a metazoan organism. In this work, we have characterised the dynamics of this histone post-translational modification during the two first cell cycles of a sea urchin embryo.

Our results show that a sharp peak of T3H3 phosphorylation coincides with mitosis in *S. granularis* early embryos. Comparing the levels of T3H3ph and the activity of the Cyclin B/CDK1 complex at different time points, we could establish that this peak of T3H3 phosphorylation precedes cleavage by about 30 min and occurs once the CDK1 activity has reached its maximal levels (Figure 1 and Figure S2). We could also show that T3H3 phosphorylation starts during the prophase in small foci associated with the chromatin (Figure 2). As the compaction of the nuclear material is already evident at this stage, our observations are thus consistent with the notion that this histone modification is not directly coupled to chromatin condensation. Later on, we observe a bright T3H3ph signal distributed along the chromosome arms, both during the prometaphase and metaphase (Figure 2 and Figure S3). Sea urchins thus differ significantly from other invertebrates, such as dipterans and tunicates, in which T3H3ph occupies the pericentromeric region during the metaphase [20,45]. In fact, our results reveal that the subcellular distribution of T3H3ph during the sea urchin first cell division is similar to that observed in mammalian somatic cells [18,19].

In summary, our findings indicate that the sea urchin embryos could become an informative experimental system to analyse the role of T3H3 phosphorylation in a developmental context. First, the observed timing and distribution of the T3H3ph signal suggests that this histone modification could accomplish similar functions in echinoderm and mammalian cells. Second, the natural synchronicity of cell division in sea urchin embryos provides a suitable context to study how the activity of the Cyclin B/CDK1 complex regulates the T3H3ph levels. To progress in this direction, we have attempted to identify the kinases responsible for T3H3 phosphorylation in *S. granularis*.

We have shown that the echinoidea species have a single Haspin homolog and that this protein contains a Ser/Thr kinase domain exhibiting all the typical structural features of its mammalian counterparts, including a well-conserved catalytic region (Figure 4). We have thus tried to block the activity of this kinase in vivo using the CHR-6494 inhibitor, a small molecule known to prevent T3H3 phosphorylation in both human and murine cells [31,32,33,41]. We found that the administration of this inhibitor does not have a direct impact on the T3H3ph levels displayed during mitosis (Figure 7). In parallel, we examined if CDK1 inhibition by Roscovitine perturbs the levels of T3H3 phosphorylation. In fact, it has been shown that Haspin activation depends on the activities of both CDK1 and the Polo-like kinase 1 (Plk1) [42,46]. If this regulatory circuit is conserved in sea urchins, we could then expect that CDK1 inhibition may indirectly impact the levels of T3H3 phosphorylation by down-regulating Haspin activity. However, the administration of Roscovitine did not elicit the presence of mitotic cells lacking a T3H3ph signal (Figure 7).

Before attempting an interpretation of these results, we should take into account two additional aspects. First, both CHR-6494 and Roscovitine consistently retard cell cycle progression, indicating that these molecules are not excluded from the egg and interfere with a biological activity necessary for cell division (Figure 6 and Figure 7). Second, our findings reveal that both drugs behave in a similar way: they delay cleavage onset (Figure 6), retard entry into the prometaphase (Figure 7) and prevent the phosphorylation of T318PP1C (Figure 5). Thus, whereas our observations are in line with the CHR-6494 anti-proliferative effects previously reported in human cancer lines and xenografted mice [41,47], they suggest that this drug could have other in vivo targets besides Haspin, such as CDK1. Our findings may thus contribute to encourage empirical studies addressing the selectivity of commonly used Haspin kinase inhibitors, like CHR-6494 or 5-Iodotubercidin (5-ITu). Indeed, a recent study has shown that 5-ITu has several in vivo off-targets and elicits a complex Haspin-independent response in mouse embryonic stem cells [48].

Putting aside the issue of drug selectivity, our results are consistent with the idea that a checkpoint system could delay the G2/M transition in sea urchin embryos until the cellular machinery is capable of implementing a robust T3H3 phosphorylation and cells can enter mitosis. According to this scenario, T3H3 phosphorylation would be tightly coupled with cell cycle progression, thus explaining why we have failed to observe cells lacking a T3H3ph signal in the prometaphase or posterior stages when Roscovitine or CHR-6494 were added to our embryo cultures. In CHR-6494 treated embryos, we cannot exclude though the possibility that the *S. granularis* Haspin kinase may be insensitive to this drug. In addition, other kinases could also phosphorylate T3H3 in the absence of Haspin activity. Given the strong phylogenetic conservation of the functional links tying Haspin activity and T3H3 phosphorylation, this latter scenario seems a priori unrealistic. However, it has been shown that the Vaccinia-Related Kinase 1 (VRK1) can also phosphorylate H3 at Thr3 and Ser10 during mitosis in human cells [49], and a VRK1 homolog is present in the genome of the sea urchin *S. purpuratus* (SPU_016006, [50]). Testing these different hypotheses will thus require the future development of specific Haspin and VRK1 inhibitors and/or targeted manipulation of their respective levels and activities during early embryogenesis.

## Supplementary Materials

The following are available online at https://www.mdpi.com/2073-4409/9/4/898/s1, Figure S1: The JY325 antibody recognises specifically the T3H3ph form in sea urchin protein extracts. Figure S2: A peak of T3H3 phosphorylation precedes cell cleavage in *S. granularis* early embryos. Figure S3: The T3H3ph signal is uniformly distributed over the chromatin during prometaphase and metaphase.
