# Supplementary material for: A Peak of H3T3 Phosphorylation Occurs in Synchrony with Mitosis in Sea Urchin Early Embryos
Source: Cells. 2020 Apr 7;9(4):898. doi: 10.3390/cells9040898 (PMC7226724; doi:10.3390/cells9040898)
Supplement: Supplementary file 1 [file cells-09-00898-s001.pdf]

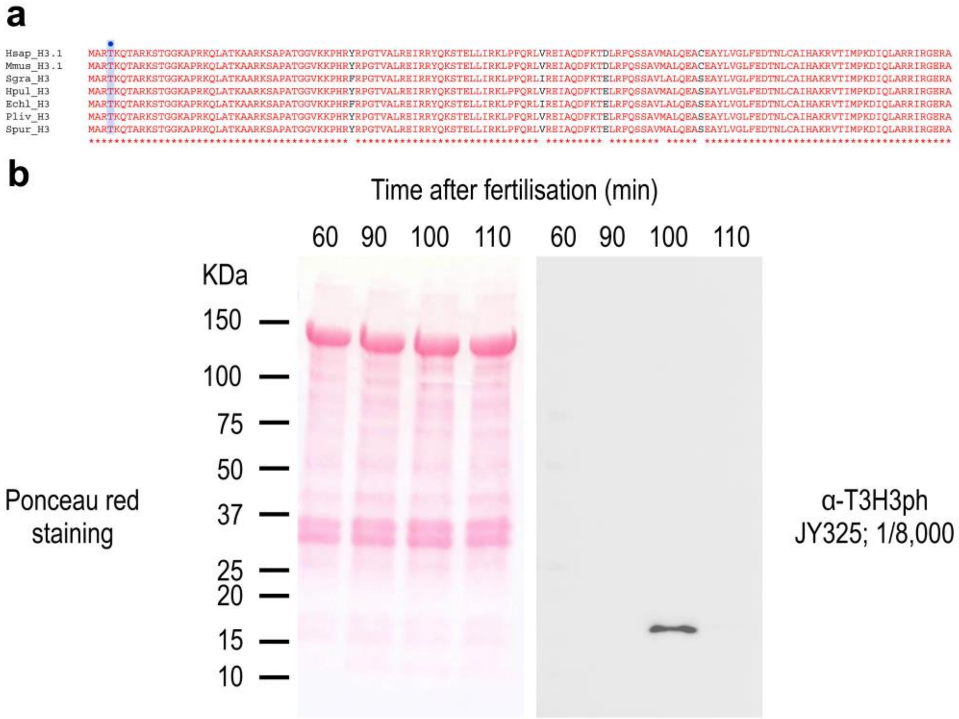

Supplementary Figure 1. The JY325 antibody recognises specifically the T3H3ph form in sea urchin protein extracts. (**a**) Clustal-W protein alignment of Histone 3 homologs in different mammalia and echinoidea species. Thr3 is shaded and labelled with a blue dot. We aligned the following sequences: Hsap, *Homo sapiens* (NP_003528.1); Mmus, *Mus musculus* (NP_038578.2); Spur, *Strongylocentrotus purpuratus* (JT107463.1); Sgra, *Sphaerechinus granularis* (GAVR01017841.1); Echl, *Evechinus chloroticus* (GAPB01032505.1); Pliv, *Paracentrotus lividus* (HACU01575671.1) and Hpul, *Hemicentrotus pulcherrimus* (IACU01073643.1). (**b**) Proteins extracts prepared with sea urchin embryos (5 μL) were separated by SDS-PAGE and visualised by Ponceau red staining (left panel). Western blot analysis of the same membrane with the T3H3ph antibody reveals a ~17 kDa single band visible in embryo extracts collected 100 min post-fertilisation (right panel).

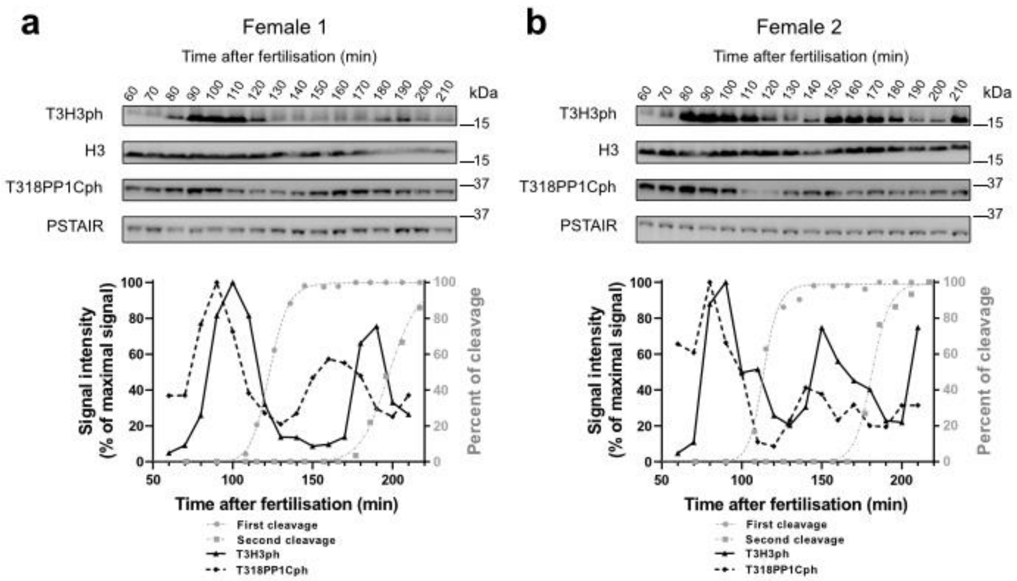

Supplementary Figure 2. A peak of T3H3 phosphorylation precedes cell cleavage in *S. granularis* early embryos. (a,b) Western blots showing the relative levels of T3H3ph, H3, T318PP1Cph and PSTAIR at different times during the first and second embryonic divisions. We examined in parallel the offspring of two different females. The graphs present a quantitative analysis of the results obtained in each separate experiment. For each lane, the T3H3ph (black triangles) and T318PP1Cph (black diamonds) signal intensity scores were respectively normalised against the values obtained for H3 and PSTAIR. Normalised values were represented as a percentage of the maximal signal observed. Graphs include as a visual reference the proportion of embryos that have completed either the first cleavage (grey dots) or the second cleavage (grey squares) at different time points.

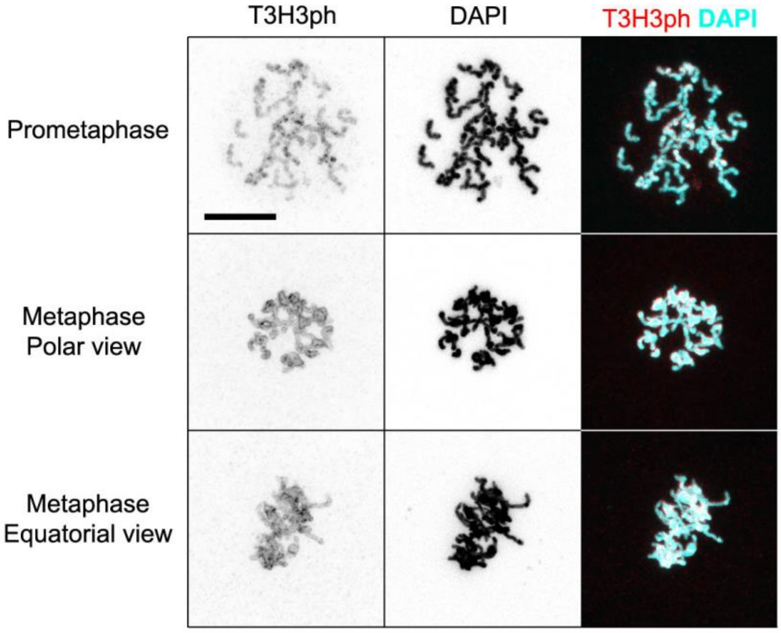

Supplementary Figure 3. The T3H3ph signal is uniformly distributed over the chromatin during prometaphase and metaphase. Images correspond to projections of confocal stacks showing the nuclear region of different sea urchin embryos during prometaphase and metaphase. Samples were immunostained for T3H3ph (inverted grey-scale in the left panels, red in merge panels) and labelled with DAPI (inverted grey-scale in the middle panels, cyan in merge panels). Scale bar, 10 μm.
